# Knowledge, Attitude, and Practice Toward Stroke Prevention and Associated Factors Among Hypertensive Patients Attending at Debre Markos and Felege Hiwot Comprehensive Specialized Hospitals, Northwest Ethiopia

**DOI:** 10.1155/nri/7379330

**Published:** 2026-07-13

**Authors:** Wuhabie Tsega, Abebe Dilie Afenigus, Setarg Ayenew Birhanie, Menberu Gete

**Affiliations:** ^1^ Department of Nursing, College of Medicine and Health Science, Debre Markos University, Debre Markos, Ethiopia, dmu.edu.et

**Keywords:** attitude, hypertension, knowledge, practice, prevention, stroke

## Abstract

**Background:**

Hypertension causes narrowing, rupture, or leakage of blood vessels. This condition causes stroke by interrupting the blood flow to the brain. The prevalence, incidence, and disability of stroke have surged due to poor knowledge, poor practices, and unfavorable attitudes toward stroke prevention. Awareness of the problem, good prevention practices, and favorable attitudes toward prevention mechanisms are the milestones to prevent stroke among hypertensive patients.

**Objective:**

This study aimed to assess knowledge, attitudes, and practices related to stroke prevention and associated factors among hypertensive patients attending at Debre Markos and Felege Hiwot Comprehensive Specialized Hospitals, Northwest Ethiopia, in 2022.

**Method:**

A cross‐sectional study design was conducted at Debre Markos and Felege Hiwot Comprehensive Specialized Hospitals, chronic illness follow‐up clinics from June 01 to July 11, 2022. A systematic random sampling technique was used to select 423 study participants. The data were collected using pretested and structured questionnaires through face‐to‐face exit interviews and chart reviews.

**Results:**

The findings of this study showed that 48.9% (95% CI: 44.0–53.8), 45.3% (95% CI: 40.4–50.2), and 44.1% (95% CI: 39.3–49.0) of the participants had good knowledge, favorable attitudes, and good practices, respectively. The factors associated with good stroke prevention knowledge included urban residence (AOR = 1.96 (1.22–3.15)), primary education (AOR = 3.67 (1.56–8.61)) or secondary education and above (AOR = 2.42 (1.47–3.99)), having a monthly income ≥ 5000 Ethiopian birr (AOR = 2.59 (1.38–4.87)), prior information about stroke (AOR = 2.29 (1.33–3.96)), and strong social support (AOR = 3.09 (1.73–5.54)). Similarly, having a monthly income ≥ 5000 Ethiopian birr (AOR = 2.05 (1.26–3.35)), moderate (AOR = 1.76 (1.03–3.03)) and strong social support (AOR = 2.27 (1.3–3.96)), and diabetes mellitus comorbidity (AOR = 5.8 (3.62–9.31)) were significantly associated with good stroke prevention practices. On the other hand, the duration of treatment (AOR = 1.68 (1.08–2.59)) was a statistically and positively associated factor with favorable attitudes toward stroke prevention.

**Conclusion:**

Nearly half of the respondents had good knowledge of stroke prevention, and four out of nine participants had favorable attitudes toward stroke prevention and good prevention practices.

## 1. Introduction

The World Health Organization (WHO) defines a stroke as “rapidly developing clinical signs of localized or global disruption of brain function lasting more than 24 h or resulting in death due to vascular origin.” [1]. Ischemic and hemorrhagic strokes are the two most common forms of stroke that disrupt cerebral blood flow [2]. Ischemic strokes account for approximately 85% of all strokes, with hemorrhagic strokes accounting for the remaining 15% [3]. Ischemic stroke occurs when blood flow to a portion of the brain is suddenly halted by a blood clot, foreign materials in the circulation, or constriction, whereas hemorrhagic stroke occurs when a blood vessel breaks apart, pouring blood into areas around neurons [4, 5].

From 1990 to 2019, the annual number of strokes and deaths due to stroke increased dramatically, especially among people over the age of 70 [6]. Between 1990 and 2019, the global stroke burden increased significantly (70% increase in incident strokes, 43% deaths from stroke, 102% prevalent strokes, and 143% disability‐adjusted life years (DALYs) lost), with the majority of deaths (86% of deaths and 89% of DALYs occurring in low‐ and middle‐income countries (LMICs)) [7].

Stroke is still the world’s second leading cause of death and the third leading cause of death and disability combined, according to the 2019 Global Burden of Disease estimates [8]. In 2019, stroke accounted for approximately 1 out of 19 deaths, and a person died of stroke every 3 min 30 s in the United States [9]. The multiple social and cognitive consequences of stroke include communication difficulties, memory loss, movement difficulties, depression, and paralysis [10]. It also has economic consequences for the sufferer, such as job loss, diminished business activity, and business failure [11].

Hypertension is the leading cause of stroke worldwide, accounting for 79.6 million DALYs or 55.5% of all stroke DALYs [6, 7]. It is also the risk factor with the strongest link to stroke, accounting for more than half of all stroke episodes worldwide [12]. Up to 98% of stroke patients in Africa have hypertension [13, 14], and it is the most powerful of the 10 major modifiable risk factors [15]. In 2019, hypertension was responsible for 53.5% of all strokes [16]. Hypertensive people are four to six times more likely to suffer a stroke than those who do not have hypertension [17].

Prevention is the primary treatment strategy aimed at reducing the morbidity and mortality related to stroke. Up to 50% of strokes can be prevented with appropriate treatment, risk factor management, and dietary and lifestyle modifications [12]. There are three stroke prevention modalities, namely, primordial, primary, and secondary. Primordial prevention strategies prevent the emergence of risk factors [18]. Primary prevention aims to reduce the risk of stroke among those who are at a risk of stroke but are asymptomatic subjects [19], while secondary stroke prevention is focused on reducing the risk of another stroke [20].

According to different studies, patients with hypertension have little understanding of stroke prevention [21–23] and lack prevention practices for stroke [24–26], while the majority have neutral attitudes toward its prevention [22]. Despite the notion that public awareness, practices, and favorable attitudes toward stroke prevention are critical for reducing stroke incidence and prevalence, there is a lack of knowledge and practices in Ethiopia, especially in the study area.

Besides, many prior studies in similar settings have focused primarily on knowledge and practice, often neglecting the attitudinal component. Furthermore, existing hospital‐based data are often from single centers, limiting their generalizability. This study, therefore, aims to fill these gaps by employing a comprehensive, reliable, and valid KAP instrument to ensure robust measurement of all three domains and generating hospital‐based data from two distinct sites (Debre Markos Comprehensive Specialized Hospital and Felege Hiwot Comprehensive Specialized Hospital) to provide a more nuanced understanding of the issue across different healthcare levels. Moreover, social support, which has not been considered as a factor in previous studies, was included in this study. Therefore, this study aimed to fill these gaps by analyzing the level of knowledge, practices, and attitudes toward stroke prevention methods among patients with hypertension at Debre Markos Comprehensive Specialized Hospital (DMCSH) and Felege Hiwot Comprehensive Specialized Hospital (FHCSH).

### 1.1. Conceptual Framework

**FIGURE 1:**
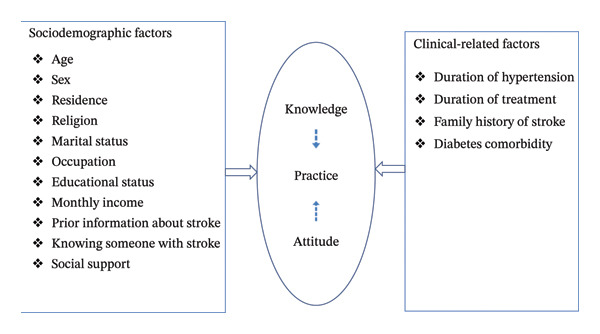
Conceptual framework of stroke prevention knowledge, attitude, and practice among hypertensive patients attending at DMCSH and FHCSH, Northwest Ethiopia, 2022.

The relationship of independent variables on the dependent variables is depicted in the conceptual framework [21, 25–31] (Figure 1).

## 2. Objectives

### 2.1. General Objective

To assess knowledge, attitudes, and practices toward stroke prevention and associated factors among hypertensive patients attending at Debre Markos and Felege Hiwot Comprehensive Specialized Hospitals, in 2022.

### 2.2. Specific Objectives

i. To determine knowledge of stroke prevention among patients with hypertension attending at DMCSH and FHCSH, in 2022.
ii. To determine attitudes toward stroke prevention among hypertensive patients attending at DMCSH and FHCSH, in 2022.
iii. To determine practices toward stroke prevention among hypertensive patients attending at DMCSH and FHCSH, in 2022.
iv. To identify factors that affect knowledge, attitudes, and practices toward prevention of stroke among patients with hypertension attending at DMCSH and FHCSH, in 2022.

## 3. Methodology

### 3.1. Study Area and Period

The study was conducted at Debre Markos and Felege Hiwot Comprehensive Specialized Hospitals from June 01 to July 11, 2022. Debre Markos and Felege Hiwot Comprehensive Specialized Hospitals are found in the Amhara Regional State, Northwest Ethiopia. These hospitals provide both inpatient and outpatient services, including chronic healthcare services. The chronic illness follow‐up clinic is an overcrowded department with regular and referral patients for chronic healthcare services. Approximately 448 and 440 hypertensive patients visited the department each month at Debre Markos and Felege Hiwot Comprehensive Specialized Hospitals, respectively.

### 3.2. Study Design

A hospital‐based cross‐sectional study design was used.

### 3.3. Population

#### 3.3.1. Source Population

All hypertensive patients who were admitted to the chronic illness follow‐up clinic at Debre Markos and Felege Hiwot Comprehensive Specialized Hospitals.

#### 3.3.2. Study Population

All hypertensive patients who visited Debre Markos and Felege Hiwot Comprehensive Specialized Hospitals chronic illness follow‐up clinics during the data collection period.

### 3.4. Eligibility Criteria

#### 3.4.1. Inclusion Criteria

All hypertensive patients who were ≥ 18 years old and came to Debre Markos and Felege Hiwot Comprehensive Specialized Hospitals chronic illness follow‐up clinics during the data collection period.

#### 3.4.2. Exclusion Criteria

Patients with hypertension who had a history of transient ischemic attack or stroke, critically ill patients, underlying severe mental illness, or were unable to provide the necessary information on their own were excluded.

### 3.5. Sample Size Determination and Procedure

#### 3.5.1. Sample Size Determination

To determine the sample size, both single and double population proportion formulas were used. For the dependent variables, a single population proportion formula was used by considering the following assumptions: proportion of good knowledge, 40.7% [30]; favorable attitudes, 50% (since no previous study); good practices, 51.7% [30]; 95% confidence interval; and 5% marginal error. The calculated sample size for knowledge is 371, whereas it is 384 for attitudes and practices.

The following assumptions were used for significantly associated factors according to calculations of the cohort or cross‐sectional sample size from StatCalc: percentage of patients who were unexposed and exposed, 82.5% and 97.1% for age, 16.5% and 37.65% for duration of hypertension, 21.48% and 52.49% for residence, and 19.1% and 55.69% for the level of education, respectively, with a 95% confidence interval, 80% power, and a 1 ratio of unexposed to exposed patients. Accordingly, sample size by Fleiss w/cc formula from StatCalc software became 160 for age, 156 for duration of hypertension, 88 for residence, and 64 hypertensive patients for level of education (Table 1).

**TABLE 1:** Summary of sample size determination for each objective.

Objectives	Proportion/*p* value	Confidence interval (CI)	Marginal error			Sample size	Reference
1. knowledge	40.7%	95%	5%			371	Study at Gondar [30]
2. Attitude	50%	95%	5%			384	
3. Practice	51.7%	95%	5%			384	Study at Gondar [30]

Factors	Power	CI	Unexposed: exposed	% outcome in unexposed	% outcome in exposed	Fleiss w/cc sample size	

1. Age	80%	95%	1	82.5	97.1	160	Study at Nigeria [26]
2. duration of hypertension	80%	95%	1	16.5	37.65	156	Study at Debre Tabor [27]
3. Residence	80%	95%	1	21.48	52.49	88	Study at Gondar [25]
4. Level of education	80%	95%	1	19.1	55.69	64	Study at Gondar [25]

Since the maximum sample size was obtained by using a single population proportion formula, the final sample size after adding a 10% nonresponse rate was 423, according to the estimated proportion of good stroke prevention practices, 51.7%, from previous study conducted in Gondar [30].

(1) i. e, n=Zα22×P1−pd2,n=1.962×0.5170.4830.052,n=38410422.4423+%=∼,

where *n* = estimated sample size, *p* = proportion of good practices for stroke prevention, and *d* = margin of error.

#### 3.5.2. Sampling Procedure

**FIGURE 2:**
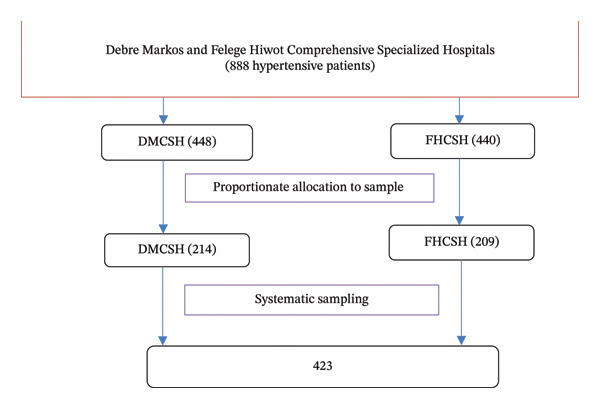
Sampling procedure at DMCSH and FHCSH, Northwest Ethiopia, 2022.

A systematic sampling technique was used to select the study participants after proportionate allocation of a sample size to each hospital. The sampling interval was 2. The first case was selected within the interval by the lottery method, and it was 2. Then, the remaining eligible participants were recruited every 2^nd^ interval until the required sample was obtained (Figure 2).

### 3.6. Study Variables

#### 3.6.1. Dependent Variables

 Knowledge, attitudes, and practices toward stroke prevention.

#### 3.6.2. Independent Variables

Sociodemographic factors (age, sex, residence, religion, occupation, educational status, marital status, monthly income, prior information about stroke, knowing someone with a stroke, and social support).

Clinical factors (duration of hypertension, duration of treatment, previous history of stroke, family history of stroke, and comorbidity).

### 3.7. Operational Definitions

 
**Good knowledge:** Respondents that score more or equal to the computed mean knowledge score [26].
 
**Favorable attitudes:** Respondents that score more or equal to the computed mean attitude score [23].
 
**Good practices:** Respondents that score more or equal to the computed mean practice score [25].
 
**Social support:** Having friends and other people including family to turn to in times of need or crisis to give you a broader focus and positive self‐image [32].
 
**Regular exercise:** Three times per week for 50 min of aerobic exercise, such as walking, jogging, and swimming [33, 34].
 
**Salt reduction:** The daily recommended salt intake is not more than 2.0 g per day (equivalent to ½ teaspoon). However, if home‐prepared spices contain salt, the addition of salt should be restricted [33, 34].

### 3.8. Data Collection Procedure

Six BSc holder nurses for data collection and two BSc nurses for supervision who were not employees of the study hospitals were selected to reduce possible sources of bias. The data collectors approached the participants politely and respectfully and explained the purpose of the study and its possible benefits. The supervisors monitored the data collection process of the data collectors, and if any problems occurred, they attempted to solve them or contacted the principal investigator.

### 3.9. Data Collection Tool

A structured closed‐ended questionnaire was prepared according to the objectives of the study and adapted from different relevant works in the literature [24, 26, 27, 35–37]. The questionnaire was then translated to Amharic by an expert and retranslated back to English by another individual for analysis and to check for any inconsistencies. The questionnaire has six parts: sociodemographic characteristics, which included 11 items; clinical‐related factors, which included 5 questions; and stroke prevention knowledge, which included 12 items with yes, no, or do‐not‐know responses and scores of 1, 2, or 3, respectively. The scale for stroke prevention attitudes contained 10 Likert‐type items ranging from 1 = strongly disagree to 5 = strongly agree responses, and the scale for stroke prevention practices contained eight items with yes or no possible responses and scores of 1 or 2, respectively. The Oslo Social Support Scale (OSSS) contained three items ranging from 3 to 14.

The internal consistency reliability of the outcome measures was examined through a pretest with 22 hypertensive patients from Injibara General Hospital using Cronbach’s α. The specific values are 0.82, 0.69, and 0.71 for knowledge, attitude, and practice items, respectively. The content validity of the questionnaire was judged by two experts (one is an assistant professor in adult health nursing and a PhD candidate, while the other is a medical doctor). Before the interviews, the questionnaires were arranged based on the content validity index (CVI) format and sent to them via email for evaluation. Based on their responses, item content validity index (I‐CVI) scores were calculated by dividing the expert agreement by the number of experts, and finally, the average of the I‐CVI scores across all items was computed. Accordingly, the content validity indices are 0.92, 0.85, and 0.94 for knowledge, attitudes, and practices, respectively, which indicates that the tool is acceptable. The client’s chart was reviewed to retrieve medical information (duration of hypertension, duration of treatment, and comorbidity).

### 3.10. Data Quality Control

Training on interview techniques was given to the data collectors one day before the data collection, and supervision of the data collectors was performed by the supervisors. Each questionnaire was checked for completeness and missed values, and incomplete questionnaires were omitted from the analysis. Pretesting in 5% (22) of the sample was performed by the principal investigator at Injibara General Hospital to assess the content and to correct unclear and vague issues on the questionnaire. Additional adjustments in the sequence and wording of the questionnaire were made based on the results of the pretest. The selected and trained supervisors supervised the data collectors on a daily basis for completeness and consistency of the completed questionnaires. In addition, the data were thoroughly cleaned and carefully entered into the computer before the beginning of the analysis.

### 3.11. Data Processing and Analysis

The collected data were checked for completeness, and the responses were coded and entered into the computer using the EpiData version 4.4 statistical package. The data were cleaned for inconsistencies and then analyzed using SPSS version 25 statistical software. Since the data were normally distributed, mean values and standard deviations were calculated. Other descriptive statistics such as frequencies and percentages were also used. Graphical presentations such as tables and bar graphs were used to present the findings of the study. Crude odds ratios (CORs), 95% confidence intervals (CIs), and *p*‐values < 0.25 were used to present the results of the bivariable analysis. All variables with a *p*‐value < 0.25 were entered into a multivariable logistic regression to assess the association between the independent and dependent variables. A multivariable logistic regression model with a backward likelihood ratio method was used to assess factors associated with stroke prevention knowledge, attitudes, and practices, and a *p*‐value < 0.05 indicated statistical significance. Multicollinearity was checked with the variance inflation factor, and its values were less than 5. Hosmer and Lemeshow goodness of fit was tested to check model fitness, and a model had *p*‐values of 0.167, 0.806, and 0.774 for knowledge, attitudes, and practices, respectively, which is > 0.05.

### 3.12. Ethical Consideration

Ethical approval was obtained from Debre Markos University Health Science College Research and Ethical Review Committee (Ref. No: HSC/R/C/Ser/PG/Co/204/11/14). Before the beginning of the data collection, a permission letter was provided to the two hospitals’ administrative bodies for data collection. At the time of data collection, both written and informed verbal consent were obtained from the participants to confirm whether they were willing to participate. There is no direct benefit from participating in this study. However, the result of the study will be helpful for improving the patients’ knowledge, attitudes, and practices toward stroke prevention. Those not willing to participate were given the right to do so. Coding was used to eliminate names and other personal identification of respondents throughout the study process to ensure participant confidentiality.

## 4. Results

### 4.1. Sociodemographic Characteristics of the Study Participants

Out of 423 patients with hypertension who were planned to be included in the study, 415 were interviewed for a response rate of 98%. Among the respondents, half of them were males. The respondents’ mean age was 53.12 years, with a standard deviation (SD) of ±16.166 years, and more than half (59.5%) of them were older than 50 years. The majority of respondents, 69.9%, were urban dwellers. Three hundred and fifty‐five (85.5%) of participants were Orthodox Christianity followers (Table 2).

**TABLE 2:** Sociodemographic characteristics of hypertensive patients at Debre Markos and Felege Hiwot Comprehensive Specialized Hospitals, Northwest Ethiopia, 2022 (*n* = 415).

Variables	Category	Frequency (%)
Age	< 50 years	168 (40.5)
	≥ 50 years	247 (59.5)

Sex	Male	209 (50.4)
	Female	206 (49.6)

Residence	Urban	290 (69.9)
	Rural	125 (30.1)

Religion	Orthodox	355 (85.5)
	Muslim	46 (11.1)
	Protestant	11 (2.7)
	Catholic	3 (0.7)

Marital status	Single	27 (6.5)
	Married	316 (76.1)
	Divorced	36 (8.7)
	Widowed	36 (8.7)

Educational status	Unable to read and write	166 (40.0)
	Able to read and write (have no formal education)	72 (17.3)
	Primary education	40 (9.7)
	Secondary education	54 (13.0)
	Diploma and above	83 (20.0)

Occupational status	Government employee	104 (25.1)
	Merchant	85 (20.5)
	Farmer	96 (23.1)
	Housewife	65 (15.7)
	Retired	40 (9.6)
	Student	10 (2.4)
	Labor worker	15 (3.6)

Monthly income	< 5000 ETB	284 (68.4)
	≥ 5000 ETB	131 (31.6)

Prior information about stroke	Yes	211 (50.8)
	No	204 (49.2)

Knowing someone with a stroke	Yes	115 (27.7)
	No	300 (72.3)

Social support	Poor	162 (39.04)
	Moderate	147 (35.42)
	Strong	106 (25.54)

### 4.2. Sources of Information About Stroke

**FIGURE 3:**
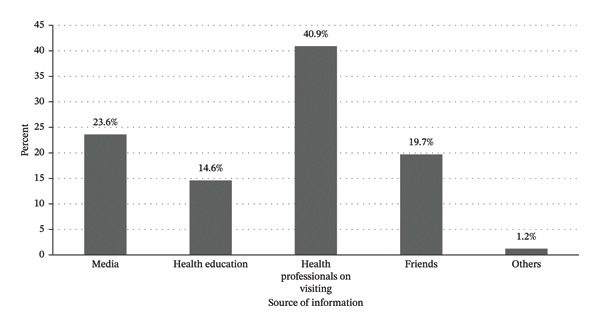
Source of information about stroke among hypertensive patients at Debre Markos and Felege Hiwot Comprehensive Specialized Hospitals, Northwest Ethiopia, 2022. Key: others = Internet, school, and newspaper.

Approximately half of the respondents (50.8%) had prior information about stroke. More than one‐third of the participants had received information from health professionals during their visits (Figure 3).

### 4.3. Clinical Characteristics

More than half of the respondents (60.5%) were diagnosed, and nearly three‐quarters of the participants (72.3%) had been followed up in the last 5 years (Table 3).

**TABLE 3:** Clinical characteristics of hypertensive patients at Debre Markos and Felege Hiwot Comprehensive Specialized Hospitals, Northwest Ethiopia, 2022 (*n* = 415).

Variables	Category	Frequency (%)
Duration of illness	< 5 years	251 (60.5)
	≥ 5 years	164 (39.5)

Duration of treatment	< 5 years	300 (72.3)
	≥ 5 years	115 (27.7)

Family history of stroke	Yes	18 (4.3)
	No	397 (95.7)

DM comorbidity	Yes	133 (32.0)
	No	282 (68.0)

### 4.4. Level of Knowledge of Stroke Prevention

The computed mean knowledge score for stroke prevention was 7.04 ± 2.84 (mean ± SD). The percentage of respondents with good knowledge to prevent the occurrence of stroke among these at‐risk populations was approximately 48.9% (95% CI: 44.0–53.8).

### 4.5. Factors Associated With Stroke Prevention Knowledge Among Hypertensive Patients

Bivariable and multivariable logistic regression analyses were used to determine factors affecting knowledge about the prevention of stroke. To adjust for potential confounders, variables that were < 0.25 in the bivariable analysis were entered into the multivariable logistic regression. The results revealed that urban residence, educational status, monthly income, prior information about stroke, and social support were significantly associated with knowledge of stroke prevention.

The odds of having good knowledge of stroke prevention were two times greater among urban residents than among those living in rural areas (AOR = 1.96; 95% CI = 1.2–3.2). Educational status was positively related to stroke prevention knowledge among hypertensive patients. Compared with patients who were unable to read and write, hypertensive patients with primary education or secondary education and above had 3.7 and 2.4 times greater odds of having good stroke prevention knowledge, respectively (AOR = 3.67; 95% CI = 1.56–8.61 and AOR = 2.42; 95% CI = 1.5–3.99). Hypertensive patients who had a monthly income of ≥ 5000 ETB were 2.59 times more likely to have good knowledge than those whose monthly income was < 5000 ETB (AOR = 2.59; 95% CI = 1.38–4.87). Patients who had prior information about stroke were 2.29 times more likely to have good stroke prevention knowledge than were those who had no information (AOR = 2.29; 95% CI = 1.33–3.96). Similarly, the odds of having good stroke prevention knowledge were three times greater among patients with strong social support (AOR = 3.09; 95% CI = 1.73–5.54) than among their counterparts (Table 4).

**TABLE 4:** Factors associated with knowledge of stroke prevention among hypertensive patients at Debre Markos and Felege Hiwot Comprehensive Specialized Hospitals, Northwest Ethiopia, 2022 (*n* = 415).

Variables	Knowledge of stroke prevention				*p* value
	Good	Poor	COR (95% CI)	AOR (95% CI)	
Age					
< 50 years	89	79	1.31 (0.89–1.95)		
≥ 50 years	114	133	1		
Sex					
Male	111	98	1		
Female	92	114	1.4 (0.95–2.07)		
Residence					
Urban	162	128	2.59 (1.67–4.02)	1.96 (1.22–3.15)^∗^	0.005
Rural	41	84	1	1	
Marital status					
Single	21	6	5.5 (1.78–16.99)		
Married	147	169	1.37 (0.68–2.77)		
Divorced	21	15	2.2 (0.86–5.65)		
Widowed	14	22	1		
Educational status					
Unable to read and write	41	85	1	1	
Able to read and write	26	40	1.35 (0.73–2.50)	1.11 (0.59–2.11)	0.746
Primary education	22	10	4.56 (1.98–10.52)	3.67 (1.56–8.61)^∗^	0.003
Secondary education and above	114	77	3.07 (1.92–4.92)	2.42 (1.47–3.99)^∗^	0.001
Occupational status					
Government employee	66	38	1		
Merchant	57	28	1.17 (0.64–2.14)		
Farmer	29	67	0.25 (0.14–0.45)		
Housewife	18	47	0.22 (0.11–0.43)		
Other	33	32	0.59 (0.32–1.11)		
Monthly income					
< 5000	107	177	1	1	
≥ 5000	96	35	4.54 (2.88–7.15)	2.59 (1.38–4.87)^∗^	0.003
Prior information about stroke					
Yes	141	70	4.61 (3.05–6.98)	2.29 (1.33–3.96)^∗^	0.003
No	62	142	1	1	
Knowing someone with a stroke					
Yes	83	32	3.89 (2.44–6.22)	1.79 (0.98–3.31)	0.060
No	120	180	1	1	
Social support					
Poor	47	100	1	1	
Moderate	66	67	2.16 (1.33–3.51)	1.53 (0.87–2.67)	0.138
Strong	89	46	4.12 (2.51–6.77)	3.09 (1.73–5.54)^∗^	0.001
Duration of illness					
< 5 years	115	136	1		
≥ 5 years	88	76	1.37 (0.92–2.03)		
Duration of treatment					
< 5 years	139	161	1		
≥ 5 years	64	51	1.45 (0.94–2.24)		
Family history of stroke					
Yes	11	4	2.98 (0.93–9.51)		
No	192	208	1		
DM comorbidity					
Yes	66	69	0.99 (0.69–1.58)		
No	137	143	1		

*Note:* 1 = reference group.

Abbreviations: AOR = adjusted odds ratio, CI = confidence interval, COR = crude odds ratio, DM = diabetes mellitus.

^∗^Statistically significant at *p* < 0.05.

### 4.6. Level of Attitudes Toward Stroke Prevention

The computed mean attitudes score for stroke prevention was 31.13 ± 2.94 (mean ± SD). Accordingly, 45.3% (95% CI: 39.3–49.0) of patients had favorable attitudes, while 54.7% (95% CI: 49.8%–59.6%) of patients had unfavorable attitudes toward stroke prevention.

### 4.7. Factors Associated With Stroke Prevention Attitudes Among Hypertensive Patients

According to the bivariable logistic regression analysis, attitudes toward stroke prevention were significantly associated with eight variables. After adjustment for potential confounders, duration of treatment was found to be significantly and positively associated with attitudes toward stroke prevention among hypertensive patients. The odds of having favorable attitudes were 1.68 times greater among patients treated for hypertension for 5 years and above than among those treated for hypertension for less than 5 years (AOR = 1.68; 95% CI = 1.08–2.59) (Table 5).

**TABLE 5:** Factors associated with attitudes toward stroke prevention among hypertensive patients at Debre Markos and Felege Hiwot Comprehensive Specialized Hospitals, Northwest Ethiopia, 2022 (*n* = 415).

Variables	Attitudes toward stroke prevention				*p* value
	Favorable	Unfavorable	COR (95% CI)	AOR (95% CI)	
Age					
< 50 years	70	98	0.78 (0.53–1.16)		
≥ 50 years	118	129	1		
Educational status					
Unable to read and write	55	71	1		
Able to read and write	32	34	1.22 (0.67–2.21)		
Primary education	19	13	1.89 (0.86–4.15)		
Secondary education and above	82	109	0.97 (0.62–1.53)		
Occupational status					
Government employee	43	61	1		
Merchant	37	48	1.09 (0.61–1.95)		
Farmer	41	55	1.06 (0.60–1.86)		
Housewife	34	31	1.56 (0.83–2.90)		
Other	33	32	1.46 (0.78–2.73)		
Knowing someone with a stroke					
Yes	99	112	1.14 (0.99–2.37)	1.46 (0.94–2.27)	0.088
No	89	115	1	1	
Duration of illness					
< 5 years	103	148	1		
≥ 5 years	85	79	1.55 (1.04–2.29)		
Duration of treatment					
< 5 years	125	175	1	1	
≥ 5 years	63	52	1.69 (1.1–2.62)	1.68 (1.08–2.59)^∗^	0.020
Previous history of stroke					
Yes	16	9	2.25 (0.97–5.22)	2.05 (0.88–4.79)	0.099
No	172	218	1	1	
Family history of stroke					
Yes	10	5	2.49 (0.84–7.43)		
No	178	222	1		
DM comorbidity					
Yes	69	66	1.41 (0.89–2.04)		
No	119	161	1		

*Note:* 1 = reference group.

Abbreviations: AOR = adjusted odds ratio, CI = confidence interval, COR = crude odds ratio, DM = diabetes mellitus.

^∗^Statistically significant at *p* < 0.05.

### 4.8. Levels of Prevention Practices for Stroke

The computed mean prevention practices for stroke patients was 5.31 ± 1.14 (mean ± SD). Accordingly, 44.1% (95% CI: 39.3–49.0) of the respondents had good practices, while 55.9% (95% CI: 51%–60.7%) of the respondents had poor practices for stroke prevention.

### 4.9. Factors Associated With Stroke Prevention Practices Among Hypertensive Patients

Like knowledge and attitudes, numerous associations were found to be significant in the bivariable analysis at a *p*‐value < 0.25. Therefore, a multivariable approach was applied to determine which factors best explained and predicted stroke prevention practices. Consequently, several independent factors, such as monthly income, social support, and having DM comorbidity, were significantly associated with good stroke prevention practices according to the multivariable analysis.

Hypertensive patients who had a monthly income of ≥ 5000 ETB were two times more likely to have good stroke prevention practices than those who had a monthly income less than 5000 ETB (AOR = 2.05; 95% CI = 1.26–3.35). Considering the respondents’ level of social support, participants who had moderate and strong social support were 1.76 and 2.27 times more likely to have good practices, respectively, than those who had poor social support (AOR = 1.76; 95% CI = 1.03–3.03 and AOR = 2.27; 95% CI = 1.30–3.96). On the other hand, patients with DM comorbidities had 5.8 times greater odds of engaging in stroke prevention practices (AOR = 5.8; 95% CI = 3.62–9.31) than those who did not have medical conditions other than hypertension (Table 6).

**TABLE 6:** Factors associated with the practices of stroke prevention among hypertensive patients at Debre Markos and Felege Hiwot Comprehensive Specialized Hospitals, Northwest Ethiopia, 2022 (*n* = 415).

Variables	Practices of stroke prevention				*p* value
	Good	Poor	COR (95% CI)	AOR (95% CI)	
Marital status					
Single	8	19	0.59 (0.2–1.7)		
Married	150	166	1.27 (0.63–2.54)		
Divorced	10	26	0.54 (0.2–1.4)		
Widowed	15	21	1		
Occupational status					
Government employee	50	54	1		
Merchant	40	45	0.96 (0.54–1.70)		
Farmer	47	49	1.04 (0.59–1.81)		
Housewife	26	39	0.72 (0.38–1.35)		
Other	20	45	0.48 (0.25–0.92)		
Monthly income					
< 5000	113	171	1	1	
≥ 5000	70	61	1.74 (1.14–2.64)	2.05 (1.26–3.35)^∗^	0.004
Social support					
Poor	51	96	1	1	
Moderate	60	73	1.55 (0.96–22.50)	1.76 (1.03–3.03)^∗^	0.040
Strong	72	63	2.15 (1.33–3.47)	2.27 (1.30–3.96)^∗^	0.004
DM comorbidity					
Yes	96	39	5.46 (3.33–5.46)	5.80 (3.62–9.31)^∗^	0.001
No	87	193	1	1	

*Note:* 1 = reference group.

Abbreviations: AOR = adjusted odds ratio, CI = confidence interval, COR = crude odds ratio, DM = diabetes mellitus.

^∗^Statistically significant at *p* < 0.05.

## 5. Discussion

The present study was conducted to assess the level of knowledge, attitudes, and practices concerning the prevention of stroke among hypertensive patients, which provides a well‐rounded investigation. In addition to being done at two sites, this study incorporates an attitudinal component using reliable and valid tool, which provides a granular understanding and a novel insight on stroke prevention. The findings of this study revealed that nearly half of the participants (48.9% (44.0–53.8)) had adequate knowledge about stroke prevention, which is higher than that reported in the findings of previous studies in Ethiopia (Debre Tabor, 24.9% [27]; two other studies in Gondar, 38.41% [25] and 40.7% [30]); and Southeast India, 11.43% [31]. The possible reason for this discrepancy may be the fact that patients may be exposed to chronic noncommunicable diseases, including stroke, which have comparable risk factors and prevention strategies. Furthermore, there was a difference in sample size and classification criteria for good and poor knowledge. Respondents who scored 75% or above on the stroke prevention knowledge‐related items were judged to have good stroke prevention knowledge in India. However, this result is lower than those of studies conducted in southern India (66.7%) [38] and Nigeria (90.8%) [26]. This may be due to sociodemographic differences in the study population. In contrast, approximately 70% of the respondents were educated in India and Nigeria.

Urban residents were two times more likely to have good knowledge of stroke prevention than were those living in rural areas. This finding is supported by research done in Debre Tabor [27] and Gondar [25]. The possible reason may be the greater accessibility of information in urban areas than in rural areas.

Another factor affecting stroke prevention knowledge among hypertensive patients was educational status. Hypertensive patients with primary education or secondary education and above had 3.7 and 2.4 times greater odds of having good stroke prevention knowledge, respectively, than did those who were unable to read and write. This was in line with the findings of many studies, according to which having a high level of education was the most significant factor for hypertensive patients toward stroke prevention in Ethiopia [24, 39], Nigeria [26], Ghana [40], Turkey [41], India [31], and China [21]. The reason could be that patients who have at least completed their elementary education may have a greater probability of being exposed to various communication media, such as magazines, books, and the internet. Furthermore, a possible explanation could be that educated people have a better understanding of their health, pay attention to individual health problems, and then take healthy measures to ensure their health.

The study showed that the odds of having good stroke prevention knowledge were 2.59 times greater among patients with a monthly income of ≥ 5000 ETB than among patients with a monthly income below 5000 ETB. This finding is in line with a study conducted in Gondar [25]. The possible reason may be the accessibility of information through media and the internet in relation to stroke, as patients with high monthly income may be able to buy and access it.

Prior information about stroke was another factor that affects knowledge of stroke prevention among hypertensive patients. Patients who had prior information about stroke were 2.29 times more likely to have good stroke prevention knowledge than were those who had no information. Having strong social support from families, friends, and the community at large was found to be a factor for stroke prevention knowledge among hypertensive patients. Patients with strong social support were three times more likely to have good stroke prevention knowledge than their counterparts. This could be due to creating a chance for smooth interaction and an area of discussion with others regarding their health problems.

In this study, 44.1% (40.4–50.2) of respondents had good prevention practices for stroke, which is consistent with a study done in Gondar (42.67%) [25]. However, this percentage is lower than that reported in other studies conducted in Gondar (51.7%) [30] and in two other studies in India (62.9% [38] and 64% [22]). This discrepancy may be due to differences in sample size and classification criteria. In a study conducted in North India, participants were considered to have good practices if they practiced 50% of the items related to stroke prevention.

Furthermore, stroke prevention practices were associated with high monthly income, moderate and strong social support, and having a concomitant disease. Those hypertensive patients with a monthly income of 5000 ETB and above were two times more likely to practice stroke prevention strategies than those who had a monthly income below 5000 ETB. This is because economical subjects have a better chance of finding information regarding the disease from various sources, which is consistent with a study done in Gondar [25].

The level of social support was the other factor in stroke prevention strategies. Good stroke prevention practices were 1.76 and 2.27 times more likely among patients with moderate and strong social support, respectively, than among patients with poor social support. This could be because interpersonal relationships help patients to engage in desired practices by providing information about their disease and may serve as a role model for good habits. Moreover, patients with diabetes mellitus and hypertension were 5.8 times more likely to practice stroke prevention than patients with hypertension alone. This could be due to the fear of late complications of the two diseases.

Approximately 45.3% (39.3–49.0) of the participants had favorable attitudes toward stroke prevention, which is lower than that reported in earlier studies in Southwest India (64.3%) [23] and South India (73.8%) [38]. A plausible reason for this discrepancy may be the small sample size. In the study conducted in South and Southwest India, 70 and 210 respondents participated, respectively, which may increase the percentage of patients with favorable attitudes toward stroke prevention if the majority of them were positively intended for items related to stroke prevention attitudes, whereas 415 hypertensive patients participated in this study.

Patients’ perceptions of stroke are crucial for better understanding and practicing preventive techniques. On the other hand, knowing the duration of treatment in hypertension management has great importance for the occurrence of hypertension‐related complications. In this study, patients who received hypertension treatment for 5 years or more were 1.68 times more likely to have favorable attitudes toward stroke prevention than those who received hypertension treatment for less than 5 years. The possible reason could be a result of repeated follow‐up contact with healthcare professionals, which can cause cognitive changes in stroke prevention strategies.

## 6. Limitations of the Study

Since the practices of patients toward stroke prevention were assessed through self‐reported practices, it may be exposed to social desirability bias and hence may increase the percentage of good practices. There may also be recall bias due to expressed practices. Clinical variables including medication adherence, BP, PR, ECG finding, and cardiac enzymes were not assessed.

## 7. Conclusion and Recommendation

### 7.1. Conclusion

Nearly half of the hypertensive patients had good knowledge of stroke prevention. Urban residency, educational status, high monthly income, having prior information about stroke, and social support were all strongly associated with good stroke prevention knowledge. Approximately four out of nine participants had good practices for stroke prevention. Monthly income, social support, and having diabetes as a comorbidity were significantly associated with stroke prevention practices among hypertensive patients. More than half of the hypertensive patients at Debre Markos and Felege Hiwot Comprehensive and Specialized Hospitals had unfavorable attitudes toward stroke prevention, and the length of treatment was found to be significantly associated with favorable attitudes toward stroke prevention.

### 7.2. Recommendations

i. Healthcare workers should disseminate information for hypertensive patients early in their appointment to enhance their knowledge, attitudes, and practices regarding stroke prevention.
ii. Policymakers and healthcare professionals should enhance stroke prevention efforts so as to reduce incidence rates and mitigate the long‐term burden of stroke‐related disability and mortality.
iii. The Woreda, Zonal, and Regional health sector can improve hypertensive patients’ knowledge, attitudes, and practices toward stroke prevention through community health campaigns and media outreach, fostering and motivating long‐term behavioral changes and performing routine screenings and access to stroke prevention programs.
iv. Families, friends, neighbors, and communities at large should support and encourage hypertensive patients to engage in stroke prevention practices.
v. Other researchers should assess patient practices related to stroke prevention using observational checklists.
vi. Researchers should conduct research on other risky population segments.

**Nomenclature**
DALYs: Disability Adjusted Life Years Lost
DMCSH: Debre Markos Comprehensive Specialized Hospital
ETB: Ethiopian Birr
FHCSH: Felege Hiwot Comprehensive Specialized Hospital
I‐CVI: Item‐Content Validity Index
OSSS: Oslo Social Support Scale

## Author Contributions

Wuhabie Tsega conceptualized and drafted the proposal and data collection tools. Wuhabie Tsega, Abebe Dilie Afenigus, and Setarg Ayenew Birhanie were involved in formal analysis, validation, and report writing. Wuhabie Tsega and Menberu Gete prepared the manuscript. All authors critically reviewed and revised the manuscript.

## Funding

No funding was received for this manuscript.

## Conflicts of Interest

The authors declare no conflicts of interest.

## Data Availability

All the data are available from the corresponding author upon reasonable request.
